# Current Landscape of Immune Checkpoint Inhibitor Therapy for Hepatocellular Carcinoma

**DOI:** 10.3390/curroncol30060439

**Published:** 2023-06-18

**Authors:** Samantha M. Ruff, Ashish Manne, Jordan M. Cloyd, Mary Dillhoff, Aslam Ejaz, Timothy M. Pawlik

**Affiliations:** 1 Department of Surgery, Division of Surgical Oncology, The Ohio State University Wexner Medical Center, Columbus, OH 43210, USA; 2 Department of Internal Medicine, Division of Medical Oncology, The Ohio State University Wexner Medical Center, Columbus, OH 43210, USA

**Keywords:** immunotherapy, immune checkpoint inhibitors, hepatocellular carcinoma, liver, cirrhosis

## Abstract

The liver maintains a balance between immune tolerance and activation in its role as a filtration system. Chronic inflammation disrupts this immune microenvironment, thereby allowing for the rise and progression of cancer. Hepatocellular carcinoma (HCC) is a liver tumor generally diagnosed in the setting of chronic liver disease. When diagnosed early, the primary treatment is surgical resection, liver transplantation, or liver directed therapies. Unfortunately, patients with HCC often present at an advanced stage or with poor liver function, thereby limiting options. To further complicate matters, most systemic therapies are relatively limited and ineffective among patients with advanced disease. Recently, the IMbrave150 trial demonstrated that the combination of atezolizumab and bevacizumab was associated with better survival compared to sorafenib among patients with advanced HCC. As such, atezolizumab and bevacizumab is now recommended first-line therapy for these patients. Tumor cells work to create an immunotolerant environment by preventing the activation of stimulatory immunoreceptors and upregulating expression of proteins that bind inhibitory immunoreceptors. ICIs work to block these interactions and bolster the anti-tumor function of the immune system. We herein provide an overview of the use of ICIs in the treatment of HCC.

## 1. Introduction

Hepatocellular carcinoma (HCC) is the most common primary liver cancer and often arises in the setting of chronic liver disease or cirrhosis. Despite advancements in cancer prevention, screening, and treatment, the incidence and mortality associated with HCC continues to increase [1]. When diagnosed early, HCC is best treated with surgical resection, liver transplantation (based on the Milan criteria or the expanded San Francisco criteria), or liver-directed therapy for small tumors [2,3]. Unfortunately, HCC often presents at an advanced stage and/or among patients with poorly preserved liver function, limiting surgical and liver directed therapeutic options. Furthermore, systemic therapies are mostly ineffective at achieving long-term survival [3].

**Figure 1:**
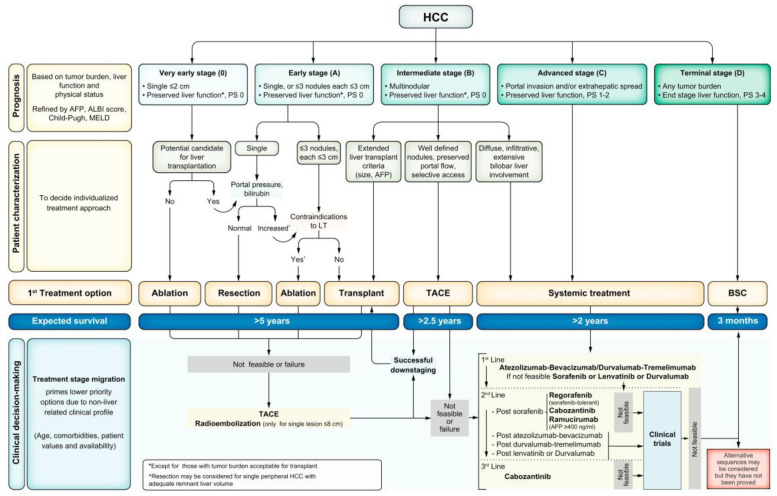
The Barcelona Clinic Liver Cancer (BCLC) system with first-line treatment recommendations based on stage of hepatocellular carcinoma (HCC). Liver function is evaluated using the Child–Pugh staging. AFP: alpha–fetoprotein, ALBI: albumin–bilirubin, BSC: best supportive care, ECOG-PS: Eastern Cooperative Oncology Group-performance status, LT: liver transplantation, MELD: model of end-stage liver disease, TACE: transarterial chemoembolization. This figure was reprinted with permission from reference [3]. Appropriate copyright permission was obtained.

Traditionally, patients with advanced HCC were treated with sorafenib, a tyrosine kinase inhibitor. The IMbrave150 trial compared sorafenib to combination atezolizumab (PD-L1 inhibitor) and bevacizumab (VEGF inhibitor) among patients with advanced HCC. Patients treated with sorafenib had a 12-month overall survival of 54.6%, whereas patients treated with atezolizumab/bevacizumab had a 12-month overall survival of 67.2% [4]. The importance of this landmark trial was reflected in a change to the Barcelona Clinic Liver Cancer (BCLC) guidelines, with atezolizumab/bevacizumab now recommended as first-line therapy for patients with advanced HCC (Figure 1) [3].

In its role as a filtration system, the liver must maintain a unique immune environment balanced between immune tolerance and activation. Chronic liver inflammation results in changes to the immune microenvironment through altered cell signaling, tissue remodeling, and an accumulation of genetic modifications. Subsequently, this disrupts the hepatic immune system’s natural anti-tumor function and plays a significant role in HCC carcinogenesis and progression. Given the recent success of the IMbrave150 trial and the unique relationship between the liver immune microenvironment and its role in cancer development, research efforts and clinical trials are now focused on identifying effective immunotherapy for HCC. Few reviews have been published that include an overview of the liver microenvironment and its role in therapy for HCC, a detailed summary of key trials for individual immune checkpoint inhibitors for the treatment of HCC, and an outline of mechanisms of resistance to therapy. We herein review current data on immune checkpoint inhibitors and their role in the treatment of HCC.

## 2. Liver Microenvironment

### 2.1. Health Liver Microenvironment

Liver sinusoidal endothelial cells (LSECs) line the lumen of the liver sinusoids. The LSECs act as a barrier for the sinusoids and allow for the exchange of plasma, nutrients, lipids, and lipoproteins while also interacting with the liver resident immune cells (e.g., natural killer cells (NK), Kupffer cells, dendritic cells, B-cells, and T-cells). The sinusoids are separated from the hepatocytes by the Space of Disse, which is where hepatic stellate cells (HSCs) and immune cells reside and where lymph fluid collects to flow into the lymphatics [5].

The liver is a complex organ that is crucial to maintaining the body’s homeostasis. Among its innumerable functions, it filters waste/foreign substances from the blood, produces bile for digestion and absorption, regulates coagulation by producing vitamin K dependent factors, stores nutrients, converts glucose to glycogen for storage and vice versa when necessary, and regulates amino acids. When filtering blood, the liver must maintain an immune system that can tolerate dietary and bacterial products that would normally stimulate an inflammatory response, but also recognize when pathogens, malignant cells, or other toxic products require intervention. Antigen-presenting cells in the liver (e.g., dendritic cells, HSCs, Kupffer cells, and LSECs) use pattern recognition receptors (PRRs) to stimulate and inhibit the immune system. After binding a microbial-associated molecular pattern (MAMP) or a damage-associated molecular pattern (DAMP), the PRRs are phagocytosed and degraded by hepatocytes and Kupffer cells. This process is accomplished without the production of inflammatory mediators that usually accompany PRR signaling in other parts of the body thereby allowing for clearance of toxins without excessive immune activation. These interactions are tightly regulated and when disrupted can be detrimental [5].

### 2.2. Microenvironment of the Chronically Inflamed Liver

Chronic inflammation can disrupt this tightly regulated system and eventually lead to carcinogenesis. Liver damage due to chronic infections (e.g., hepatitis) or excessive alcohol or fat consumption can initiate carcinogenesis. Normal physiologic events in response to liver damage, such as tissue remodeling, are driven by inflammatory mediators. For example, an increase in regulatory T-cell activity, secretion of IL-10 and TGF-β, and inhibition of antigen presentation can occur in the setting of chronic liver inflammation. Kupffer cells release reactive oxygen species and growth factors that activate HSCs to secrete matrix metalloproteinases (MMPs) to remodel the extracellular matrix (ECM). As fibrosis forms, the LSECs reduce their fenestrations and form a continuous basement membrane to protect the liver from continued toxin exposure and damage. However, this process also reduces blood flow and delivery of nutrients to the hepatocytes. To protect the liver, NK cells kill HSCs to stop production of MMPs, but a pro-inflammatory state continues to be promoted. In turn, the immune system becomes increasingly exhausted from constant activation within the liver. These exhausted T-cells have limited activity against abnormal cells. This, combined with the decreased presence of helper cytokines and suppressed T-cell proliferation, make the liver more vulnerable to the growth and expansion of abnormal cells (e.g., HCC) [5,6].

## 3. Immune Checkpoints

**Figure 2:**
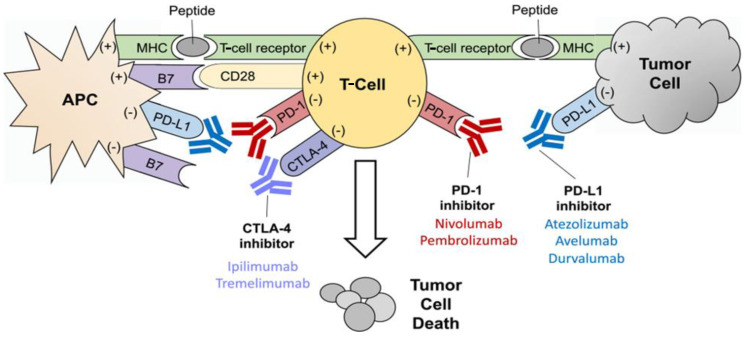
Mechanisms of T-cell activation and inhibition. T-cell activation is mediated by the interaction of the T-cell receptor with the major histocompatibility complex (MHC) and the CD28 receptor with the B7 costimulatory molecule on the antigen-presenting cell (APC). Activating interactions are noted with a plus sign (+). T-cell inhibition is mediated by the interaction of PD-L1 and PD-1, as well as CTLA-4 and B7. Inhibitory interactions are noted with a minus sign (−). Inhibitors of PD-1, PD-L1, and CTLA-4 prevent the inactivation of T-cells, thus allowing the T-cells to destroy the tumor cell more effectively. This figure is from an open-access journal and does not require copyright permission. It was reprinted from reference [10].

Immune checkpoints are membrane proteins that act as regulators of the immune system by binding to receptors on immune cells acting to inhibit or stimulate. The physiologic function of immune checkpoints is to suppress potential autoreactivity so that the immune system does not attack cells indiscriminately [7]. However, cancer cells are able to take advantage of this system by expressing proteins that bind inhibitory immunoreceptors, which can downregulate expression of proteins that bind stimulatory immunoreceptors. These mechanisms can allow cancer cells to evade the immune system [8]. Overexpression of inhibitory signals on cancer cells in the tumor microenvironment also leads to T-cell exhaustion. These T-cells have limited activity against cancer cells, diminished production of helper cytokines, and suppressed T-cell proliferation [9]. Immune checkpoint inhibitors (ICIs) are monoclonal antibodies that block these inhibitory interactions, allowing T-cells to perform their intended anti-tumor function. ICIs have the additional benefit of more-tolerable side effects versus cytotoxic chemotherapy (Figure 2) [8].

### 3.1. Cytotoxic T-Lymphocyte-Associated Protein 4 (CTLA-4)

The family of B7 ligands can stimulate or suppress the immune system by binding different T-cell receptors [11]. When B7 on antigen-presenting cells (APCs) binds the T-cell receptor CD28, it provides a second stimulatory signal and establishes a pro-inflammatory state [12]. In contrast, when B7 binds the T-cell surface receptor CTLA-4, T-cell clonal expansion is downregulated and B7 is sequestered so that it cannot bind CD28 [13]. CTLA-4 binds B7 with higher affinity and often outcompetes CD28 receptors [12]. Cancer cells use this immune checkpoint as a way to suppress and exhaust T-cells, making it a prime target for ICIs.

### 3.2. Programmed Death Ligand-1 (PD-L1)/Programmed Cell Death Protein-1 (PD-1)

PD-L1 is expressed on somatic cells in response to proinflammatory cytokines and binds to the PD-1 receptor on T-cells, B-cells, natural killer (NK) cells, myeloid-derived suppressor cells, and dendritic cells. When bound, T-cell migration, proliferation, and cytotoxin secretion are suppressed. Tumor cells can evade tumor-infiltrating lymphocytes by expressing PD-L1 and binding PD-1 to inhibit the immune system [14]. This mechanism is a particularly intriguing target for patients with HCC, since overexpression of PD-1 and PD-L1 has been demonstrated in chronically inflamed livers [15,16].

## 4. Immune Checkpoint Inhibitors

Traditionally, based on data from the SHARP trial, advanced HCC was treated systemically with sorafenib [17]. Unfortunately, overall prognosis was still poor for patients treated with sorafenib. As the driving mechanism of carcinogenesis has become better-understood, cancer therapy has shifted from a focus on cytotoxic chemotherapy to immunotherapy and targeted therapy for a personalized approach. Even with the “same” histologic HCC diagnosis, different patients have a varied response to systemic therapies, suggesting that targeting specific genetic aberrations or unique driving mechanisms of cancer can provide more effective treatment. Given the unique immune microenvironment of the liver, leveraging the immune system to treat HCC has great appeal. To this point, the landmark IMbrave150 trial demonstrated that combination atezolizumab and bevacizumab was associated with improved overall and progression free survival versus sorafenib among patients with advanced HCC [4]. More recently, ICIs have been explored in the treatment of HCC as a monotherapy or in combination with other systemic therapies.

### 4.1. Tremelimumab and Durvalumab

Tremelimumab is a CTLA-4 inhibitor. The first trial to establish its potential efficacy was a phase II trial in 20 patients with inoperable HCC and chronic hepatitis C (43% had Child–Pugh B liver disease) [18]. Treatment with tremelimumab resulted in a disease control rate of 76.4% and a partial response rate of 17.6%. Unfortunately, the partial response lasted only 3.6 months, 9.2 months, and 15.8 months in three patients. Tremelimumab resulted in a decrease in viral load and had an acceptable safety profile. Given the low response rate, combination therapy was explored in subsequent clinical trials in hopes of increasing efficacy.

A randomized phase II trial examined different combinations of tremelimumab and durvalumab (PD-L1 inhibitor) in 332 patients with advanced HCC [19]. Patients received either durvalumab monotherapy, tremelimumab monotherapy, combination tremelimumab and durvalumab every four weeks, or a priming dose of tremelimumab with durvalumab every four weeks. While a response was noted in all four treatment arms, the tremelimumab priming dose with durvalumab demonstrated the greatest efficacy. This combination therapy had an objective response rate of 24% and median overall survival of 18 months. All four cohorts had acceptable safety profiles at the end of the study. The recently published HIMALAYA phase III trial assigned 1171 patients with advanced HCC and no previous treatment to receive either durvalumab monotherapy, sorafenib, or priming dose of tremelimumab with durvalumab every four weeks [20]. Median overall survival with combination tremelimumab/durvalumab was 16.4 months versus 13.8 months with sorafenib. At 36 months, combination tremelimumab/durvalumab had an overall survival of 30.7% versus 24.2% in the durvalumab monotherapy cohort, and 20.2% in the sorafenib cohort. In October and December 2022, combination tremelimumab/durvalumab was approved for patients with unresectable HCC in the United States and Europe, respectively [21]. Currently, combination tremelimumab/durvalumab is considered an appropriate alternative first-line therapy to atezolizumab/bevacizumab for patients with advanced disease according to the BCLC guidelines [3]. The ongoing phase III EMERALD-3 randomized trial is evaluating the combination of transarterial chemoembolization (TACE), durvalumab, and tremelimumab with or without Lenvatinib versus TACE alone in patients with locoreginal HCC not amenable to curative surgery, transplant, or ablation (NCT05301842).

### 4.2. Nivolumab and Ipilimumab

Nivolumab was the first PD-1 inhibitor approved in 2017 as second-line therapy by the Food and Drug Administration for the treatment of HCC [22]. This approval was based on data from the Checkmate 040 and 459 trials. Checkmate 040 was a phase I/II multicenter trial that included patients with Child–Pugh B liver disease and advanced HCC [23]. Half of the patients in this study were previously treated with sorafenib and the other half were sorafenib-naïve. The objective response rate was 12% and the disease control rate was 55%. The median duration of response was 9.9 months. This study established that nivolumab had an acceptable safety profile in patients with underlying liver impairment. The Checkmate 459 trial compared nivolumab to sorafenib among patients with advanced HCC and no previous systemic therapy (previous surgical or locoregional treatment was allowed) [24]. While there was not a significant difference in overall survival between the two cohorts, the results were somewhat biased, since patients who progressed on sorafenib crossed over to the nivolumab cohort. Nivolumab had a lower rate of grade 3 and 4 adverse events. Retrospective studies comparing nivolumab monotherapy and sorafenib have demonstrated similar findings, with no clear difference in survival; nivolumab has been associated with a lower toxicity, however [25].

The combination of tremelimumab and durvalumab has been demonstrated to be more effective than tremelimumab monotherapy. Overall, ICI monotherapy seems to have more-limited benefits in the treatment of HCC compared with combination therapies. Combination therapy may be more effective by targeting multiple immune checkpoints and overcoming immune cell exhaustion on multiple fronts. However, it is still unclear if the success of combination immunotherapy is secondary to a synergistic or additive effect. A common combination regimen to treat HCC is a PD-1 inhibitor and CTLA-4 inhibitor. One of the first trials to demonstrate the potential combination ICI therapy for HCC was a trial that treated 148 patients with HCC who had previously been on sorafenib to receive nivolumab and/or ipilimumab (CTLA-4 inhibitor) at different doses and intervals [26]. The objective response rate was 31% with combination therapy versus 15% in nivolumab monotherapy. At 24 months, overall survival was 40%. Based on these data, nivolumab/ipilimumab therapy was approved as second-line treatment after sorafenib for patients with advanced HCC. There is currently a randomized phase II trial evaluating the efficacy of neoadjuvant nivolumab monotherapy versus nivolumab/ipilimumab among patients with HCC who are eligible for surgical resection (NCT03222076) [27]. Preliminary data analysis of 20/27 patients (7 did not undergo surgery) demonstrated a progression-free survival of 9.4 months among patients treated with nivolumab monotherapy cohort versus 19.53 months among individuals treated with nivolumab/ipilimumab cohort. Interestingly, no patient who had a major pathologic response (≥70% necrosis on resected tumor) experienced a recurrence at a median follow up of 26.8 months; in contrast, roughly one-half of patients who did not have a major pathologic response developed recurrent disease. This study demonstrated that neoadjuvant ICI was safe among patients with resectable HCC. In addition, the data suggested that a major pathologic response to ICIs may provide insight into prognosis among patients undergoing resection of HCC.

Ongoing studies are also evaluating the use of immunotherapy in combination with locoregional treatments. In a phase I randomized trial, patients with advanced HCC received stereotactic body radiation therapy (SBRT) followed by either nivolumab or nivolumab/ipilimumab [28]. Median overall survival was 41.6 months versus 4.7 months in the SBRT/nivolumab/ipilimumab and SBRT/nivolumab cohorts, respectively. These data further demonstrate that combination immunotherapy likely may have a synergistic effect to prolong survival.

HCC often arises in the setting of a chronically diseased liver. As such, even after curative treatment, the underlying environment that promotes carcinogenesis is still present. In turn, 5-year survival after surgical resection or ablation of ranges only from 50% to 80%, and many patients experience a recurrence [3]. As a result, research has focused on whether neoadjuvant or adjuvant immunotherapy may help to prevent recurrence or new disease formation. In a phase I trial, 15 patients with high-risk HCC were treated with nivolumab and carbozantinib (tyrosine kinase inhibitor) [29]. On final pathology after surgical resection, four patients had a >90% pathologic response and one had a complete pathologic response. In a phase II trial, 27 patients with resectable HCC were given either nivolumab or nivolumab/ipilimumab (CTLA-4 inhibitor) as neoadjuvant therapy and adjuvant therapy for two years [27]. No patient had a recurrence at two years and roughly 30% of patients had a partial pathologic response. There are several ongoing trials investigating the use of neoadjuvant ipilimumab and nivolumab (NCT03222076, NCT 03682276), neoadjuvant carbozantinib and nivolumab (NCT03299946), and adjuvant nivolumab (Checkmate 9DX NCT03383458).

### 4.3. Pembrolizumab

Pembrolizumab is a well-known PD-1 inhibitor that is effective in treatment of several cancers. In the Keynote-224 trial, 169 patients with HCC who progressed on or could not tolerate sorafenib were treated with pembrolizumab [30]. Treatment with pembrolizumab resulted in a partial or complete response in 17% of patients, stable disease in 44%, and progressive disease in 33% of patients. Given the efficacy and acceptable safety profile in this trial, pembrolizumab was approved for use in HCC. Unfortunately, the Keynote-240 trial, which randomized 413 patients to receive either pembrolizumab or a placebo after progressing on sorafenib, was less successful [31]. While patients in the pembrolizumab cohort demonstrated an objective response rate of 18.3% versus 4.4% in the placebo cohort, there was no difference in overall survival between the two cohorts. Pembrolizumab has some anti-tumor activity against HCC, but as a monotherapy has not demonstrated an improvement in survival. Given these results, many European societies do not endorse pembrolizumab for HCC [32]. It is also important to note that a strong objective response rate does not always correlate with improved overall survival. It is important that patients are followed to a primary end-point of either recurrence-free/progression-free or overall survival to truly understand the clinical efficacy of these treatments. Pembrolizumab is currently being investigated in clinical trials for use as neoadjuvant or adjuvant therapy (NCT03337841, NCT03867084).

Given conflicting results as a monotherapy, studies have explored the combination of pembrolizumab and Lenvatinib (tyrosine kinase inhibitor). In a phase I trial, 104 patients with unresectable HCC (most patients without any prior systemic therapy) were treated with pembrolizumab and Lenvatinib [33]. Median progression-free survival was 9.3 months and median overall survival was 22 months. There were grade 3 adverse events in 67% of patients, but the events were all manageable. Wu et al. evaluated 71 patients with unresectable HCC who received Lenvatinib and pembrolizumab [34]. For 62% of patients, this regimen was used as first-line therapy and for 38% of patients as second-line therapy (after targeted therapy or nivolumab). The objective response rate and disease control rate were 34.1% and 84.1%, respectively. Prior nivolumab failure and Child–Pugh class B were both associated with poor overall survival on multivariable analysis. In a separate study, Chen et al. reported on 170 treatment-naïve patients with unresectable HCC who received pembrolizumab and Lenvatinib with or without hepatic artery infusion pump (HAIP) therapy. The median overall survival was 17.7 months in the HAIP/pembrolizumab/Lenvatinib cohort versus 12.6 months in the pembrolizumab/Lenvatinib cohort [35]. There is an ongoing randomized phase III clinical trial, LEAP-012, that is evaluating the use of TACE with or without combination Lenvatinib/pembrolizumab in patients with intermediate-stage HCC (NCT04246177) [36].

### 4.4. Atezolizumab

Atezolizumab, a PD-L1 inhibitor, has proven efficacy for HCC. The IMbrave150 trial randomized 501 patients with advanced HCC to receive either atezolizumab/bevacizumab or sorafenib [4]. This study demonstrated an overall survival at 12 months of 67.2% in the atezolizumab/bevacizumab cohort versus 54.6% in the sorafenib cohort. Median progression-free survival was 6.8 months in the atezolizumab/bevacizumab cohort compared with 4.3 months in the sorafenib cohort. This landmark trial resulted in a change to the BCLC guidelines, as atezolizumab/bevacizumab is now recommended as first-line therapy over sorafenib for advanced HCC. Of note, the trial only included patients with preserved liver function and many patients with HCC have underlying liver dysfunction. In the extended follow-up study to the IMbrave150 trial, atezolizumab/bevacizumab still maintained a clinically meaningful survival benefit [37]. Retrospective, real-world studies have compared outcomes among patients with HCC who were treated with atezolizumab/bevacizumab relative to previous patients who had been treated with sorafenib or Lenvatinib; these data demonstrated a survival advantage for atezolizumab/bevacizumab treatment [38,39,40,41]. These studies included patients with liver dysfunction and noted that liver function was better-preserved with the immunotherapy regimens. The IMbrave050 trial is currently evaluating the efficacy of adjuvant atezolizumab/bevacizumab compared with active surveillance among patients with resected or ablated HCC (NCT04102098). There is also an ongoing trial evaluating the benefit of adding ipilimumab to atezolizumab/bevacizumab to treat patients with HCC (TRIPLET trial, NCT05665348).

### 4.5. Other Immune Checkpoint Inhibitors

PD-1/PD-L1 and CTLA-4 inhibitors are only successful in a fraction of patients with HCC. This is likely due to the heterogeneity of HCC tumor antigens among different patients, as well as different tumors within the same patient. T-cell immunoglobulin mucin-3 (TIM-3), lymphocyte activation gene-3 (LAG-3), and B- and T-lymphocyte attenuator (BTLA) are promising targets currently being studied in ongoing trials [42,43,44,45,46,47]. These trials include evaluation of cobolimab (TIM-3 inhibitor) and dostarlimab (PD-1 inhibitor, NCT-3680508), and relatlimab (LAG-3 inhibitor, NCT04567615, NCT05337137, NCT04658147).

## 5. Mechanisms of Resistance

Despite some success in clinical trials, ICIs are only effective in about 30–40% of patients with HCC. Primary resistance refers to the tumor not initially responding to ICIs, while acquired (or secondary) resistance occurs when patients have disease recurrence or progression after an initial response. There are many potential mechanisms for resistance and several studies have focused on trying to identify patient or tumor factors that may help guide clinicians as to which ICIs will be most effective in patients [48].

### 5.1. Primary ICI Resistance

**Figure 3:**
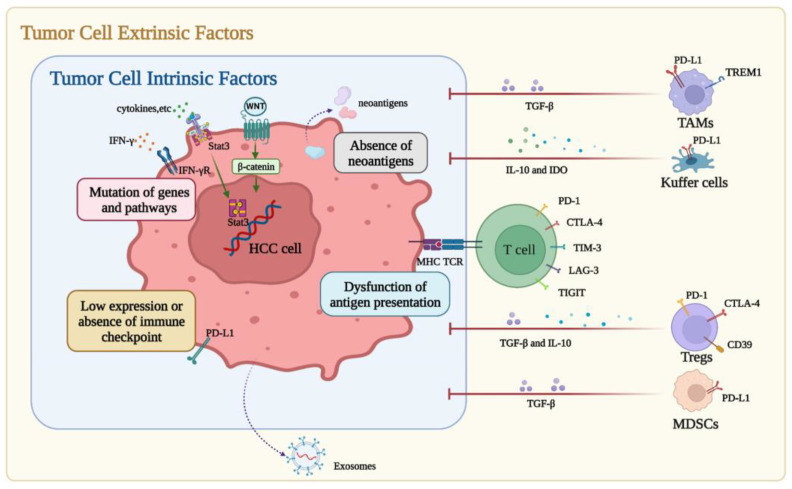
Primary resistance to immune checkpoint inhibitors (ICIs) in hepatocellular carcinoma (HCC). Factors of primary resistance can be divided into tumor cell intrinsic factors and tumor cell extrinsic factors. The former includes low or absent immune checkpoint expression, absence of neoantigens, dysfunction of antigen presentation, and mutations of genes or pathways, while the latter includes immunosuppressive cells and molecules and HCC-derived exosomes. This figure was reprinted with permission from reference [49]. Appropriate copyright permission was obtained.

Primary resistance can be divided into tumor cell intrinsic and extrinsic factors (Figure 3) [49]. Immunohistochemical staining for immune checkpoint expression (e.g., PD-L1) has demonstrated some predictive value to identify which patients will respond to ICIs in lung, breast, and esophageal cancer, but has not demonstrated the same predictive value in HCC [49]. One of the leading mechanisms of primary resistance to ICIs is a low tumor mutational burden (TMB). A high TMB results in increased neoantigens and potentially makes the tumor more immunogenic. In studies of melanoma and non-small cell lung cancer, a strong response to ICIs has been observed among patients with a high TMB. These studies also demonstrated that patients with a high TMB treated with ICIs had improved overall survival compared with individuals with a low TMB [50,51,52]. In a study of 755 patients with advanced HCC, the median TMB was four mutations/Mb and only 0.8% of patients exhibited a high TMB [53]. Low TMB may be a key reason for primary ICI resistance among patients with HCC.

Another mechanism of primary resistance is the dysfunction of neo-antigen presentation. Tumor cells can decrease their expression of neo-antigens through various mechanisms (e.g., hypermethylation of genes to suppress expression of antigens) or can acquire genetic mutations that alter their ability to even present antigens [54,55,56,57]. One example of this is a mutation in the β2-microglobulin gene that leads to reduced major histocompatibility complex (MHC) I expression and subsequently decreased antigen presentation and evasion of the immune system [54,55,56,57]. HCC tumors often contain a high copy-number alteration burden, which can lead to changes in chromosome structure and loss of genes that are necessary for antigen presentation [58]. In one study, patients with HCC who responded to ICIs had an upregulation in MHC-II molecules, implying increased neo-antigen presentation [59]. These data further suggest that there is a relationship between antigen presentation and resistance to ICIs.

In addition to intrinsic tumor factors (such as TMB or dysfunctional antigen presentation), extrinsic factors in the immune microenvironment can contribute to ICI resistance. The tumor microenvironment includes a variety of cells and signaling molecules that can create a suppressed immune environment allowing for the propagation of cancer and resistance to ICIs [48]. Specifically in HCC, only about 25% of HCCs have markers of an inflammatory response on gene-expression profiling [60]. Other studies have noted that HCC cells secrete exosomes that can upregulate PD-L1 expression on macrophages to help evade the immune system, induce NK cell exhaustion, or impair function of T-cells [49]. Translational research is key to moving the field forward. Data from clinical trials including data on the response to therapy and TMB or specific pre-existing genetic aberrations in HCC tumors should help inform experiments in the laboratory. In the future, a greater understanding of underlying mechanistic pathways may help to identify which patients will benefit the most from ICIs.

### 5.2. Secondary (Acquired) ICI Resistance

Unfortunately, mechanisms of acquired ICI resistance are poorly understood, especially in HCC. Tumor heterogeneity is likely the driving mechanism behind acquired resistance. While PD-1/PD-L1 and CTLA-4 are commonly found in the tumor microenvironment, expression of additional immune checkpoints, such as TIM-3 or LAG-3, have also been noted [61]. Targeting these additional immune checkpoints in combination with PD-L1 and CTLA-4 inhibitors may help to reverse immune exhaustion and overcome acquired resistance [62]. In a similar fashion, ICIs are effective against ICI-sensitive cells, but the remaining population of cells may contain mutations that facilitate resistance to ICI therapy. Surviving cells can clone themselves to make up a majority of the tumor. Clinically, these patients will initially respond to ICIs, but ultimately progress [63]. One way to potentially overcome this is through profiling of the tumor and using combination therapy to target the tumor heterogeneity. Again, this is best accomplished through strong translational research. The “bench to bedside” relationship is a two-way street. Data from the laboratory informs clinical trials, but also data from clinical trials can be used to inform experiments in the lab. Understanding the underlying genetic aberrations of ICI-sensitive versus ICI-resistant HCC cells may help overcome secondary resistance. Genetic profiling of the tumor may help identify which patients will develop resistance, which may assist in early intervention to add other therapies or changing the regimen accordingly.

## 6. Conclusions and Expert Opinion

HCC is a rare, aggressive primary liver cancer that often presents at an advanced stage. When diagnosed early, HCC can be treated with liver transplantation, surgical resection, or liver-directed therapy. Unfortunately, HCC often arises in the setting of liver dysfunction and presents at an advanced stage. Systemic therapies are limited and for the most part ineffective at improving long-term survival. The liver’s immune environment is a balance between tolerance and activation. Chronic inflammation disrupts this homeostasis and leads to immune cell exhaustion and fibrosis. This environment primes the liver for the development of HCC. Given the unique environment and relative lack of effective systemic therapeutic options, research has shifted focus to the role of immunotherapy in the treatment of HCC. Regulatory immune checkpoints are used by cancer cells to evade the immune system. Immune checkpoint inhibitors are monoclonal antibodies that block these inhibitory interactions and help to reinstate the natural anti-tumor function of T-cells. Clinical trials have demonstrated that ICIs are more effective in HCC when used in combination with other ICIs or targeted therapy (e.g., tyrosine kinase inhibitors). Currently, combination atezolizumab/bevacizumab or durvalumab/tremelimumab are considered first-line therapy for advanced HCC based on these clinical trial data. Choosing between these two regimens can be difficult. One strategy is to identify patients at risk for specific adverse events. For example, gastrointestinal bleeding is a well-known risk of bevacizumab. Therefore, patients were screened and treated for esophageal varices prior to enrollment on the IMbrave150 trial. As such, durvalumab/tremelimumab may be preferred in patients who cannot tolerate VEGF inhibitor therapy. Another strategy is to identify patient- or tumor-specific factors that may be associated with response to specific therapeutic regimens. Patients with portal vein tumor thrombosis were included in the IMbrave150 trial (atezolizumab/bevacizumab), but excluded from the HIMALAYA trial (durvalumab/tremelimumab). Given the rarity of these tumors, it is crucial that these patients are referred and treated in clinical trials and that large institutions work together to expedite accrual. These approaches can help identify which therapy will improve overall survival for specific subsets of patients, whether it is an established first-line combination therapy or a new therapy being tested.

Despite some success, only about 30%–40% of patients respond to combination ICI therapy. The mechanisms of primary and secondary resistance are still being elucidated and strategies to overcome these obstacles require more in-depth research. The benefit of combination therapy is that it may help reverse immune exhaustion and overcome acquired resistance. Data from clinical trials should be used to inform experiments in the laboratory and vice versa through strong translational research. This approach will help better identify the underlying mechanisms for success or failure of combination therapies and define which patients will benefit the most from these therapies. ICIs are a promising field in the treatment of HCC, but significant work remains to improve outcomes for patients with HCC.

## Data Availability

Not applicable.
